# A critical review on *In Vivo* and *Ex Vivo* models for the investigation of *Helicobacter pylori* infection

**DOI:** 10.3389/fcimb.2025.1516237

**Published:** 2025-05-14

**Authors:** Shwetlaxmi Patil, Songmin Yu, Renitta Jobby, Vinothkannan Ravichandran, Sohinee Sarkar

**Affiliations:** ^1^ Amity Institute of Biotechnology, Amity University Maharashtra, Mumbai, India; ^2^ Murdoch Children’s Research Institute, Parkville, VIC, Australia; ^3^ Department of Pediatrics, University of Melbourne, Parkville, VIC, Australia; ^4^ Amity Centre of Excellence in Astrobiology, Amity University Maharashtra, Mumbai, India; ^5^ Center for Drug Discovery and Development (CD3), Amity Institute of Biotechnology, Amity University Maharashtra, Mumbai, India

**Keywords:** *Helicobacter pylori*, animal models, antimicrobial resistance, gastritis, peptic ulcer disease, gastric cancer, gastric organoids

## Abstract

*Helicobacter pylori* is a stomach-dwelling bacterium with a crude global prevalence of nearly 45% in adults and 35% in children and adolescents. Chronic *H. pylori* infection and the resulting inflammation are major causes of gastritis, peptic ulcer disease and gastric cancer. Since its discovery in 1982, various animal models have been proposed to recreate the specific pathophysiological interactions between *H. pylori* and the human host. These infection models have been instrumental in dissecting the key drivers of *H. pylori* colonization, persistence and mediators of host immune responses. However, a comprehensive understanding of the molecular triggers for malignant transformation of the gastric mucosa is still lacking. Vaccine development in this area has stalled, as promising candidates identified through animal studies have failed in advanced human clinical trials. Currently, *H.* pylori eradication is heavily reliant on different antimicrobial agents. As with other bacterial pathogens, the growing antimicrobial resistance in *H. pylori* remains a major challenge, making eradication therapy increasingly complex and prolonged, over time. Recent drug approvals have mostly been for newer combinations of conventional antibiotics and proton pump inhibitors. Thus, the development of novel treatments and innovative models are crucial for advancing the drug development pipeline. This review encompasses the development and recent advances in animal and non-animal models of *H. pylori* gastric infection and its applications in investigating novel therapeutics and vaccine candidates.

## Introduction

The microaerobic *Helicobacter pylori* is a Gram-negative, spiral rod-shaped bacterium that predominantly colonizes the human gastric mucosal surface and is associated with acute and chronic gastritis, peptic ulcers, and other upper gastrointestinal disorders (Aihara et al., 2014; Chen et al., 2024). Recent estimates of *H. pylori* colonization show a crude global prevalence of nearly 45% in adults and 35% in children and adolescents (Chen et al., 2024). Despite the high prevalence, in 80%-85% of cases, the bacteria’s existence is unrelated to any clinically symptomatic disease (Elbehiry et al., 2023). However, individuals with *H. pylori-*associated chronic gastritis have a 1%-3% chance of developing gastric cancer (Uemura et al., 2001) and a 10%–20% chance of developing peptic ulcers (Peek, 2008). The World Health Organization (WHO) has designated *H. pylori* infection as a class I carcinogen due to its ability to cause gastric adenocarcinoma (stomach cancer) and mucosa-associated lymphoid tissue (MALT) lymphoma (Vogiatzi et al., 2007). Presently, gastric cancer ranks as the fifth most common cancer worldwide and the fifth leading cause of cancer-related deaths (Bray et al., 2024).

**Figure 1:**
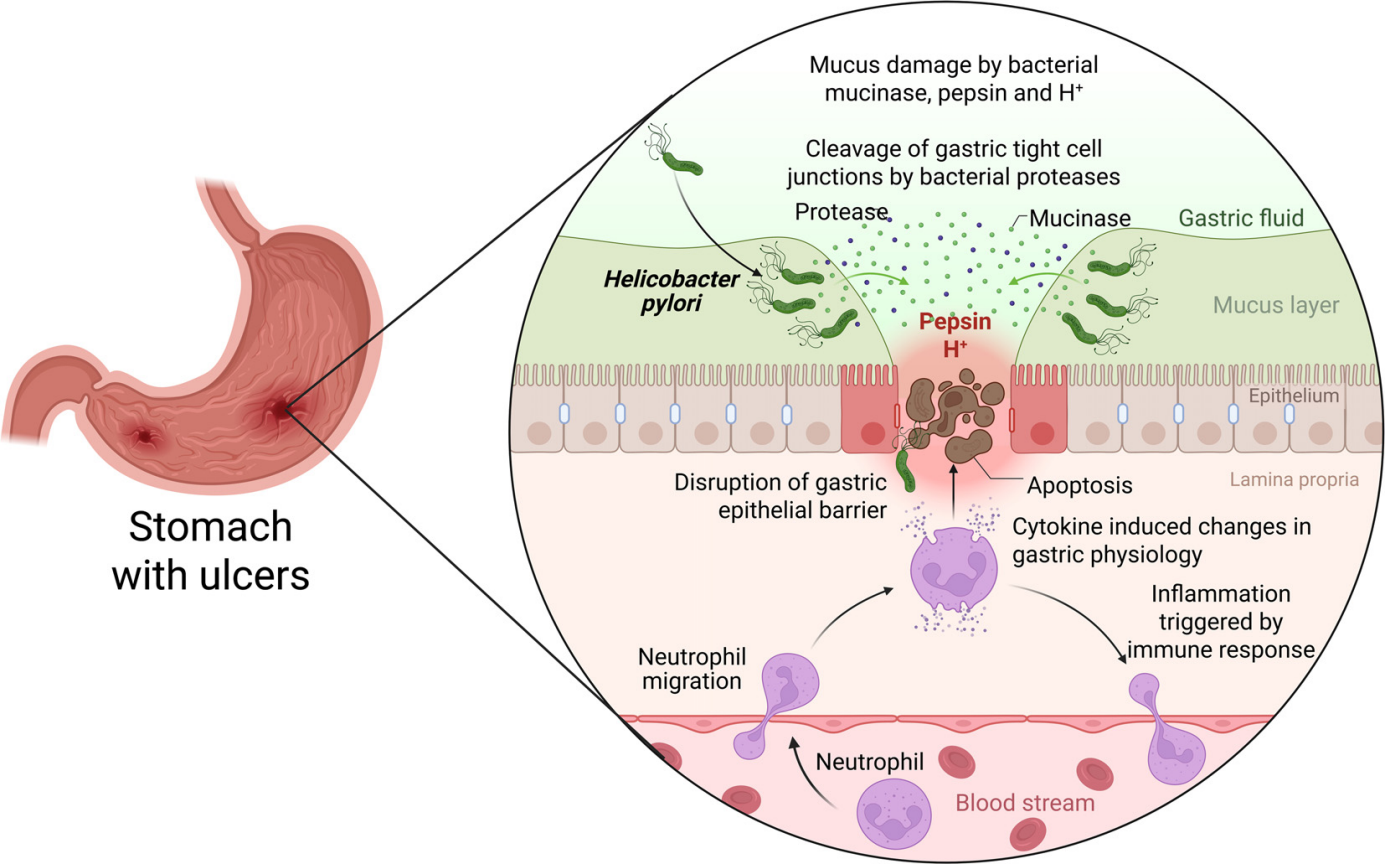
Pathogenesis of *H. pylori* infection within the gastric epithelium. Three main stages of *H. pylori* pathogenesis can be studied: i) bacterial attachment to and colonization of the stomach mucosa; ii) the host’s immune response and *H. pylori’s* immune-evasive mechanisms; and iii) the pathological consequences of chronic infection (Elbehiry et al., 2023) *Created with BioRender*.

The prevalence of *H. pylori* varies between and within countries, but it is generally estimated to range from 30% to 50% in developed countries and from 60% to 80% in underdeveloped regions (Chen et al., 2024; Poddar, 2019). These estimates are based on stratified variables like geography, age, socioeconomic status, and ethnicity, which result in significant regional and national variations. In India, the prevalence of *H. pylori* infection has been reported to be relatively high, with studies indicating that up to 80% of children under the age of 10 may be infected (Poddar and Yachha, 2007). The high prevalence of *H. pylori* infection in India can be attributed to factors such as poor sanitation, overcrowding, and lack of access to clean water (Thirumurthi and Graham, 2012). *H. pylori* is typically acquired very early on in childhood via oral-oral, fecal-oral or iatrogenic routes of transmission (Brown, 2000). Having evolved with the human host for thousands of years (Linz et al., 2007), these bacteria demonstrate a remarkable ability to survive in the harsh acidic environment of the stomach, localizing within the gastric mucosal layer during chronic infection (**Figure 1**) and potentially persisting for the host’s lifetime. *H. pylori* have an arsenal of different colonization and virulence factors that facilitate its persistence within the gastric niche. Bacterial factors such as urease production, chemotactic motility, and the capacity to adjust to the changing gastric environment, all contribute to its ability to survive in the stomach for decades (Yamaoka, 2010). However, the more severe disease states are associated with presence of the cytotoxin-associated gene A (*cagA*) and vacuolating cytotoxin A (*vacA*) genes, both which encode for corresponding polymorphic cytotoxins that are injected and secreted by *H. pylori* and contribute significantly to its pathogenesis (Jones et al, 2010). *H. pylori* can further produce diverse classes of lytic enzymes, including lipases, phospholipases, and proteases, which degrade gastric mucus by altering its viscosity and hydrophobicity (Asante et al., 1997; Celli et al., 2009). The compromised mucus barrier allows *H. pylori* to closely associate with the gastric epithelium while making the latter more susceptible to gastric acid. It has recently been proposed that biofilm development also contributes to chronic colonization (Carron et al., 2006; Cole et al., 2004). 

## *H. pylori* and the gastric precancerous cascade

Gastric cancer is the most serious pathological consequence of *H. pylori* infection, although exact steps leading up to it are poorly understood. While gastric cancer can be broadly divided into diffuse and intestinal types, it is the latter that is most commonly associated with *H. pylori* infection (Uemura et al., 2001; Hanada and Graham, 2014). The progression of chronic gastritis towards the development of the gastric ulcers and eventually, gastric cancer, is associated with mucosal pH variations (Brawner et al, 2014) and distinct, sequential changes within the tissue. This precancerous process takes place, presumably over several decades, before any clinical presentation of cancer. First delineated by Pelayo Correa in 1992 (Correa, 1992), specific stages of the precancerous cascade have been refined over the years. The ‘Correa Cascade’ describes the transition of the gastric mucosa from chronic active gastritis → chronic atrophic gastritis → intestinal metaplasia → dysplasia and ultimately, invasive carcinoma (Correa, 2013). This process is triggered by *H. pylori* infection and sustained by the resultant chronic inflammation. It was initially proposed that eradication of *H. pylori* till the stage of intestinal metaplasia can successfully halt the progression towards gastric cancer. However, more recent evidence suggests that while *H. pylori* eradication is still the most important method for preventing gastric cancer globally, its effect on established gastric intestinal metaplasia is limited (Cheng et al., 2023; Zhu et al., 2024). Thus, early and successful eradication of *H. pylori* is critical for the prevention of gastric cancer.

## Current challenges in treatment of *H. pylori*: rise in antimicrobial resistance

The sole and current therapy for *H. pylori* infection in humans is the use of antibiotics (Suzuki et al., 2022). The triple and quadruple therapy regimens are used as the first line of defense against *H. pylori* infection depending on local resistance profiles and treatment guidelines. According to worldwide recommendations, the most commonly used first-line treatment for *H. pylori* infection involves a ‘triple therapy’ that includes a proton pump inhibitor (PPI) combined with two broad spectrum antibiotics (amoxicillin, clarithromycin, levofloxacin, and metronidazole) for 7 to 14 days (Chey et al., 2024). However, because of the prevalence of antibiotic resistance, the eradication rates of therapy have declined to <80% in many countries (Jiang et al., 2022; Aumpan et al., 2023). The addition of Bismuth to the PPI-antibiotic combination constitutes the ‘quadruple therapy’ regimen that is being increasingly used as a first line treatment in areas with high resistance (Chey et al., 2024).

Antimicrobial resistance is acknowledged as a significant public health issue with worldwide implications, particularly considering that the rate of advent of multidrug resistant bacteria has vastly outpaced and the slow discovery of novel antibiotics (Ortiz et al., 2019; Aumpan et al., 2023). Antimicrobial resistance may be more prevalent in developing nations than in high-income countries, even though statistics are scarce in low- and middle-income countries (Jaka et al., 2018; Ortiz et al., 2019). Despite concerted stewardship efforts, antimicrobials are increasingly being consumed worldwide, often without appropriate prescription or compliance (Zarauz et al., 2022; Ventola, 2015). Global antimicrobial usage grew by at least 35% in the last ten years, with only a handful of countries responsible for 75% of this growth (Collaborators, 2024). In many nations with significant antimicrobial usage, the high rates of self-medication and the availability of antimicrobials over the counter are of particular concern (Ortiz et al., 2019). In addition to variations in antibiotic resistance rates, there can be differences in the prevalence of the infection within and across different topographical regions within a country (Jiang et al., 2022).

Within the context of *H. pylori* eradication therapy, the results of a recent meta-analysis study released in 2018 show that the rates of primary and secondary resistance to levofloxacin, metronidazole, and clarithromycin have already reached alarming levels (>15%) in nearly all WHO areas (Shah et al, 2021). This has led to the failure rate of triple therapy rising to more than 20% in many regions of the world (Shah et al, 2021). Considering the poor prognosis of gastric cancer and the significant morbidity and costs associated with earlier stages of *H. pylori* pathologies (such as peptic ulcer disease), there is a critical need to create novel therapeutic approaches to combat antimicrobial resistance in *H. pylori*. Even though *H. pylori* has been excluded from the most recent iteration of WHO’s priority pathogen list for urgent drug development, the report acknowledges the increasing complexity of treatment and associated adverse effects and failure rates, which necessitates a renewed focus by researchers and drug developers, alike.

Here in this review, we have discussed various animal models, including knock-out and transgenic models, available for the study of various stages of *H. pylori* pathophysiology (**Figure 1**). We have further emphasized the *ex vivo* models such as gastric and intestinal organoids along with their advantages and limitations that can be a valuable addition to the resources that can be utilized for the development of novel therapeutics and vaccine candidates. 

## Animal models

**Figure 2:**
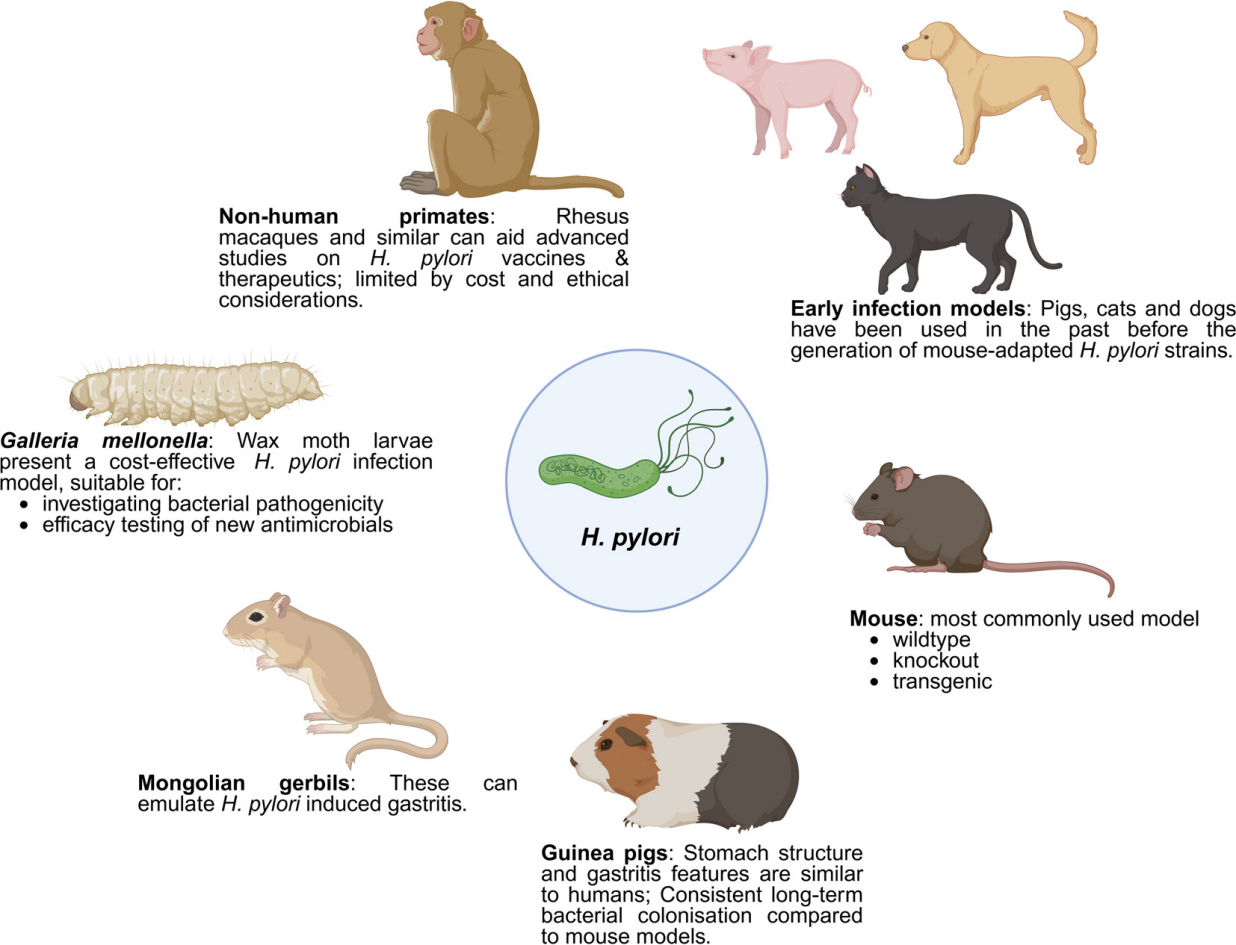
Various *in vivo* models for the study of *H. pylori* pathogenesis. This figure illustrates various animal models employed in the study of *H. pylori* infection and its pathogenesis. Early infection models, including pigs, cats, and dogs, were initially used before the development of mouse-adapted *H. pylori* strains. Mice remain the most commonly used model, available in wildtype, knockout, and transgenic varieties. *Mongolian gerbils* are effective in emulating *H. pylori*-induced gastritis, while guinea pigs, with stomach structures and gastritis features similar to humans, allow for consistent, long-term bacterial colonization studies. Non-human primates, such as rhesus macaques, are useful for advanced studies on *H. pylori* vaccines and therapeutics, despite limitations due to cost and ethical considerations. Wax moth larvae (*Galleria mellonella*) present a cost-effective infection model, suitable for investigating bacterial pathogenicity and testing new antimicrobials. Created with BioRender.

While animal models have been instrumental in identifying some of the key features of *H. pylori* pathogenesis that underpins chronic gastritis and the development of ulcers, the exact triggers for gastric carcinogenesis remain under investigation (Ansari and Yamaoka, 2022). The natural history of infection in animals is unknown, and *H. pylori* does not readily infect the gastric mucosa of animals, despite the fact that it is well adapted to colonize the human stomach (Go, 2002). Investigations during early infection in humans are often hindered by the natural pathophysiology of the disease as gastric cancer takes decades to develop due to the complicated interactions between *H. pylori* and the stomach epithelium (Correa and Piazuelo, 2008). The pathogenesis of *H. pylori* infection and the immunological responses brought on by this bacterium have been difficult to ascertain due to the close co-evolution of this pathogen with its human host. However, animal models must be used in order to fully understand the role of host’s microenvironment in various *H. pylori*-induced disease states, including gastric cancer. These models have indeed been very helpful in understanding the pathophysiology of *H. pylori* colonization (Peek, 2008) with an emphasis on the comprehension of host immune responses and the natural history of infection (**Figure 2**). Animals like pigs, rodents, mice, Mongolian gerbils, and guinea pigs have all been mentioned as potential reservoirs for *H. pylori* (Peek, 2008). Thus, appropriate animal models must be used to enable a thorough understanding of the role of the host’s microenvironment that sustains and drives *H. pylori*-induced inflammation and gastric cancer. Although these animal models have been beneficial in understanding the host, bacterial, and environmental variables involved in gastritis and gastric carcinogenesis, no single model stands as the definitive standard for mimicking natural human infection (**Table 1**).

**Table 1:** Summary of different *in vivo* models used to study *H. pylori* pathogenesis with its advantages, limitations and applications in research.

Animal Model	Characteristics/Advantages	Research Focus	Limitations	References
Early models (gnotobiotic pigs, dogs and cats)	Used in early *H. pylori* research prior to the establishment of mouse infection models	Instrumental in identifying critical bacterial virulence factors such as urease and motility Early characterization of lymphofollicular gastritis	Significant immune differences from humans; economic and logistical challenges. Lower colonization levels, acute infection symptoms, and not widely adopted.	(Fox et al., 1995; Handt et al., 1995; Radin et al., 1990; Eaton et al., 1989; Krakowka et al., 1987)
*Galleria mellonella* (wax moth larvae)	Quick determination of *in vivo* toxicity. Cost-effective model for *H. pylori* virulence factors.	Screening clinical *H. pylori* strains. Discriminating between virulent and avirulent strains. Studying pathogenic mechanisms.	Incomplete recapitulation of mammalian infection; supplementary to more physiologically relevant models.	(Giannouli et al., 2014; Krzyżek et al., 2025; Ochoa et al., 2021)
Mouse models	Widely used for modelling different stages of pathogenesis and genetic disorders. Humanized models available for physiological similarity. Vast resource of molecular tools for genetic manipulation and immunological studies.	Testing *H. pylori* mediated gastric pathology Investigation of novel antimicrobials and phytochemicals. Limited insight into severe *H. pylori* illnesses. Investigating immune responses and vaccination strategies.	Milder gastritis and slow disease progression; variability in bacterial virulence; complex pathogenic mechanisms.	(Lee et al., 1997; Dey et al., 2021; Jiang and Yu, 2017; Menheniott et al., 2016; Ohnishi et al., 2008; Sawai et al., 1999; Stair et al., 2023)
Mongolian gerbils	Mirror *H. pylori-*induced human symptoms and diseases. Cost-effective model for studying probiotics.	Validating vaccines and mutation analysis. Studying gastritis, Cag T4SS, and microbiota changes. Testing proton pump inhibitor therapy.	Varied cancer development timelines; lack of molecular resources.	(Guo et al., 2019; Isobe et al., 2012; Jeremy et al., 2006; Kuo et al., 2013; Lin et al., 2020)
Non-human primates	Ideal for physiological studies due to similarities with humans. Chronic gastritis observed in pigtailed macaques and rhesus monkeys.	Testing late-stage vaccine candidates against *H. pylori* prior to human clinical trials Genetic analysis of complex physiological traits.	High cost, time and labor-intensive, ethical concerns, and limited widespread use.	(Semrau et al., 2017; Solnick et al., 2003, Solnick et al, 2006, Solnick et al, 2001)
Guinea pigs	Useful model for studying antral gastritis caused by *H. pylori.* Similar gastric anatomy and physiology to humans.	Modeling *H. pylori-*induced stomach diseases. Studying pathogenesis and host immune response. Examining IL-8 expression and epithelial cell changes.	Limited widespread use despite anatomical and pathophysiological similarities to humans.	(Shomer et al., 1998; Sturegård et al., 1998; Tomaszewska et al., 2024)

### Early animal models

Gnotobiotic piglets were probably the first animals to undergo successful infection with *H. pylori* (Lambert et al., 1987; Krakowka et al., 1987; Eaton et al, 1989). While these animals have been instrumental in studying the role of urease and motility in the mediation of *H. pylori* infection induced pathology, key immune features observed in this model can vary significantly from humans. Despite its early applications, researchers have moved away from using this model, likely due to economic limitations and the need for specialized breeding and experimental facilities.

Beagle dogs, both gnotobiotic pups and conventional animals, have been investigated for experimental *H. pylori* infection. Gnotobiotic pups, infected at 7 days of age, showed successful *H. pylori* colonization for at least a month post-infection, albeit at a lower level compared to humans (Radin et al., 1990). Gastric lesions were observed upon gross examination with microscopic evidence of immune cell infiltration into the gastric lamina propria. Antibodies specific to *H. pylori* were developed in conventional beagle dogs that were infected between four and six months of age and observed for up to 24 weeks (Rossi et al., 1999). Animals with an acute infection experienced vomiting and diarrhea. This was followed by polymorphonuclear cell infiltration and subsequent development of gastritis with epithelial changes linked to the development of MALT lymphoma in humans. Even though the pathophysiology of *H. pylori* infection in dogs closely resembles that of humans, this model has not been adopted more widely. Similarly, there are not many reported studies on feline infections. The initial descriptions of feline *H. pylori* infection demonstrated the development of lymphofollicular gastritis (Fox et al., 1995; Handt et al., 1995). According to a vaccination study, cats that received the urease antigen orally were protected against the *H. pylori* challenge (Handt et al., 1995). However, there have not been wider studies with this host species other than reports of isolation and characterization of non-*H. pylori Helicobacter* isolates.

### *Galleria mellonella* larvae

Insects can be employed to quickly determine *in vivo* toxicity and efficacy of novel antimicrobial compounds, which makes it possible to conduct more targeted mammalian testing. *Galleria mellonella* is a wax moth present throughout the world and its larvae have been used as animal model in several research studies for over two decades (Tsai et al, 2016). The larvae of *G. mellonella* are being utilized more frequently as miniature hosts to study the pathogenesis and virulence components of many bacterial and fungal human pathogens from humans despite the fact that they do not recapitulate all elements of mammalian infection. This has the following benefits: i) adaptation to the human physiological temperature (37°C), ii) presence of a well-characterized phagocytic system, and iii) availability of a comprehensive transcriptome and immune gene repertoire. Several pathogens, including *Pseudomonas aeruginosa, Staphylococcus aureus, Acinetobacter baumannii, Klebsiella pneumoniae*, and *Campylobacter jejuni*, have been shown to be pathogenic in *G. mellonella* larvae (Tsai et al, 2016). Due to their susceptibility to *H. pylori* infection and relatively lower thresholds of legal or ethical considerations, *G. mellonella* larvae have been proposed as a simple *in vivo* model for the study of *H. pylori* virulence factors and pathogenic pathways (Giannouli et al., 2014). The experimental paradigm that has been developed can be helpful for screening a relatively large number of clinical *H. pylori* strains and for correlating the disease state of patients with the virulence of these strains (Giannouli et al., 2014). Recently this model was utilized to demonstrate biofilm formation by multidrug resistant *H. pylori* strain during its exposure to stress caused by clarithromycin (Krzyżek et al., 2025). The *G. mellonella* larval infection model recapitulates important aspects of *H. pylori* pathophysiology and is cost-effective in comparison to mammalian infection models. While it could be useful for the initial evaluation of the impact of *H. pylori* virulence parameters factors on particular cellular functions, it is unable to completely substitute the well-established and more physiologically relevant *in vivo* models in the analysis of the complex pathogenic mechanisms underlying *H. pylori*-related human disease (Giannouli et al., 2014; Ochoa et al., 2021) and the efficacy of therapeutics (Almeida Furquim de Camargo et al., 2020). In summary, the *G. mellonella* model may lessen reliance on mammalian infection models based on its ability to differentiate between virulent and non-virulent *H. pylori* isolates, determine putative genes associated with virulence through genome-wide association studies and identify novel molecular targets for antimicrobial therapy.

### Mouse models

Mice have been a crucial model organism to study different human infectious diseases and genetic disorders. The predominantly used wildtype mouse strains in *H. pylori* infection are C57BL/6 and BALB/c, followed by less commonly used Swiss albino, ICR and Kunming mice (Ansari and Yamaoka, 2022; Wei et al., 2019; Kimura et al., 1998). These models are used to study the effects of *H. pylori* colonization and to test the efficacy of different antimicrobials, phytochemicals, and probiotics. The bacterial strain used to infect these mice presents another important facet to the infection as mice do not get colonized by *H. pylori* as readily as humans (Salama et al, 2013). It is interesting to note that before *H. pylori* infection models were developed, mouse infection models and vaccination trials extensively employed a feline *Helicobacter* isolate named *H. felis*. *H. felis* lacks the *cag* pathogenicity island and produces severe atrophic gastritis in C57BL/6 mice, but only mild disease in BALB/c mice (Zhang and Moss, 2012). In 1997, Lee et al., introduced the first standardized mouse infection model of *H. pylori* with the Sydney strain SS1 derived from an individual with duodenal ulcers (Lee et al., 1997). The original strain was *cag* ^+^ *vac* ^+^ (PMSS1) but underwent modification during experimental infection to yield the type 4 secretion system (T4SS) deficient SS1 strain widely used in infection studies nowadays. The parental PMSS1 strain is often used to study the effect of a functional cagPAI system (Arnold et al., 2011) although it has been reported that this genetic island is susceptible to genetic rearrangement and disruption leading to variability in bacterial virulence features (Dyer et al., 2018). Overall, *H. pylori* infection in wildtype mice frequently results in milder gastritis or slowly progressive illnesses, and these models offer less insight into the toxicity of clinical *H. pylori* strains. Furthermore, while these wildtype mice infected with *H. pylori* and *H. felis* develop lymphocytic gastritis, they generally do not advance to more severe conditions such as peptic ulcers or stomach cancer (Zhang and Moss, 2012).

To compensate for the deficiencies of the wildtype mouse models, several knockout and transgenic mice have been developed over the years. These have incrementally paved the way for the identification of critical host factors implicated in *H. pylori* pathogenesis and the development of gastric cancer. Some examples are reviewed below.

*H. pylori* infection-induced gastric inflammation is largely mediated by IFN-gamma and double-knockout mice have been demonstrated to allow longer colonization by strains that are unable to infect wildtype mice (Sawai et al., 1999). Chronic infection is typically associated with significantly lower inflammation in this model which has been used for many vaccine studies. Similar observations have been made in mice deficient in TNF-alpha signaling (Yamamoto et al., 2004).

*H. pylori* infection has a synergistic effect on the development of gastric cancer in individuals with IL-1β gene polymorphisms with the most severe gastric pathology being observed in patients with both host and bacterial high-risk genotypes (El-Omar et al., 2000). IL-1 receptor knockout mice have lower gastritis scores and associated pathology markers such as nitric oxide production (Huang et al., 2013). Gastrokine-2 is an anti-inflammatory protein produced in the gastric epithelium and its deletion drives premalignant gastric inflammation and tumor progression in mice that is accelerated by *H. pylori* infection (Menheniott et al., 2016). Furthermore, GKN-2 expression is progressively lost during the progression of gastric cancer, and it plays a causal role in its development (Chung Nien Chin et al., 2020). During early *H. pylori* infection, Fas-antigen mediated apoptosis depletes gastric parietal and chief cells which are then replaced by metaplastic glandular lineages resistant to Fas-apoptosis. This has been modelled in Fas antigen–deficient (*lpr*) mice that develop invasive stomach lesions post *H. pylori* infection (Cai et al., 2005).

Transgenic mice are genetically engineered mice that harbor gene insertions from different species. Humanized insulin/Gastrin (INS-GAS) transgenic mice are frequently used to model stomach cancer as they have high circulating levels of pancreatic gastrin (Jiang and Yu, 2017). These mice spontaneously develop atrophic gastritis and intestinal metaplasia which then progress to corpus-centric cancer. PMSS1 infection in IN-GAS mice results in invasive carcinoma whereas SS1 infection causes only dysplasia (Lofgren et al., 2011; Stair et al., 2023), thus demonstrating the importance of bacterial factors in determining disease progression. Transgenic expression of *H. pylori* CagA in mice has been shown to induce gastrointestinal and hematopoietic neoplasms (Ohnishi et al., 2008).

### Mongolian Gerbils

Mongolian gerbils are small rodents that show similar symptoms to humans, such as appetite and weight loss, and recount many attributes of *H. pylori*-induced gastric colonization, inflammation, ulcers, and cancers (Noto et al., 2016). After it was discovered that Mongolian gerbils can mirror several characteristics of *H. pylori*-induced human stomach inflammation and cancer, these rodents captured considerable interest and attracted a lot of attention, particularly for vaccine studies (Jeremy et al., 2006; Guo et al., 2019; Lv et al., 2014; Noto et al., 2016; Wu et al., 2008). However, the timeline for cancer development varies greatly between studies and appears to be contingent on the infecting *H. pylori* strain. Gerbil infection by a modified *H. pylori* strain with an inducible T4SS has demonstrated that while an infectious trigger can be instrumental for cancer development, cancer progression does not necessarily depend on the persistent presence of the infectious agent (Lin et al., 2020). Comparative genomic studies in this model have shown that the genomes of three strains recovered from infected gerbil stomachs showed mutations in *babA*, *tlpB*, and *gltS* genes, all of which are linked to host adaptation (Suzuki et al., 2019).

This model has also been used in the study of probiotics in *H. pylori* eradication (Kuo et al., 2013; Zheng et al., 2024). Probiotics have been shown to inhibit *H. pylori* growth, adhesion, and the production of virulence factors *in vitro*. In the gerbil infection model, probiotics have been demonstrated to prevent the colonization of *H. pylori* (Zheng et al., 2024; Kuo et al., 2013; Isobe et al., 2012). This model has also demonstrated that the presence of *H. pylori* and related inflammation can alter microbiome composition in the non-inflamed regions of the gastrointestinal tract (Heimesaat et al., 2014). While the Mongolian gerbil infection model has been instrumental in dissecting the critical determinants mediating host-pathogen interactions during *H. pylori* infection, the lack of molecular resources has stymied its wider application within the research community.

### Guinea pigs

The guinea pig stomach structure is akin to that of humans with a dietary requirement for vitamin C like humans and other primates. *H. pylori* infection in this model, first described in the late 1990’s, produces an inflammatory response driven by IL-8 secretion by gastric epithelial cells (Sturegård et al., 1998; Kleanthous et al, 1998). Infections with either the rodent adapted SS1 strain or clinical *H. pylori* isolates from gastric biopsies were able to establish lasting colonization and induce seroconversion in infected animals. Chronic infection was associated with the development of antral gastritis and lymphoid follicles like that of *H. pylori*-infected humans. Epithelial cell proliferation as evidenced by the presence of a large population of Ki67-positive gastric cells, is readily observed in this model (Gonciarz et al., 2019). This model has been used in a recent study delineating the role of *H. pylori* in driving metabolic syndrome (Tomaszewska et al., 2024). Despite the similarities with human gastric anatomy and *H. pylori* related pathophysiology, this model has not been widely used.

### Non-human primates

Non-human primates, such as macaques, can be a suitable model for *H. pylori* infections due to their physiological and anatomical similarities with humans. Rhesus macaques *(Macaca mulatta)* acquire *H. pylori* in the stomach mucosa and experience chronic gastritis (Solnick et al., 2003, Solnick et al., 2001) and glandular atrophy, the precursor to stomach cancer (Dubois et al., 1999). However, it remains unclear if macaques naturally harbor *H. pylori* and act as a reservoir in wildlife or whether they acquire the bacteria upon contact with humans, after capture (Solnick et al., 2003, Solnick et al., 2006). Non-human primates are mostly used in testing vaccines against *H. pylori* (Lee, 2001). The genetic and physiological parallels between primates and humans make this model appealing and practical for research on *H. pylori*-related gastritis, but the high cost, time and labor requirements along with ethical considerations prevent its widespread use in *Helicobacter* research (Barnhill et al, 2016).

## Non-animal (*ex vivo*) models

The preceding sections discuss various *in vivo* infection models that facilitate extended bacterial exposure in the host, proving crucial in identifying key factors driving *H. pylori* pathogenesis. However, these animal models do not precisely mirror the pathophysiological responses observed in humans (**Table 1**). Additionally, the large numbers of animals required have raised concerns about animal welfare and cost (Burkitt et al., 2017). *In vitro* studies using the widely available gastric cancer cell lines, on the other hand, do not have the complex cellular and architectural details of the intact gastric epithelium (Wagner et al., 1994; Birkness et al., 1996; Saberi et al., 2018). However, they do provide a more controlled environment for studying the intricate details of how hosts and pathogens interact.

**Figure 3:**
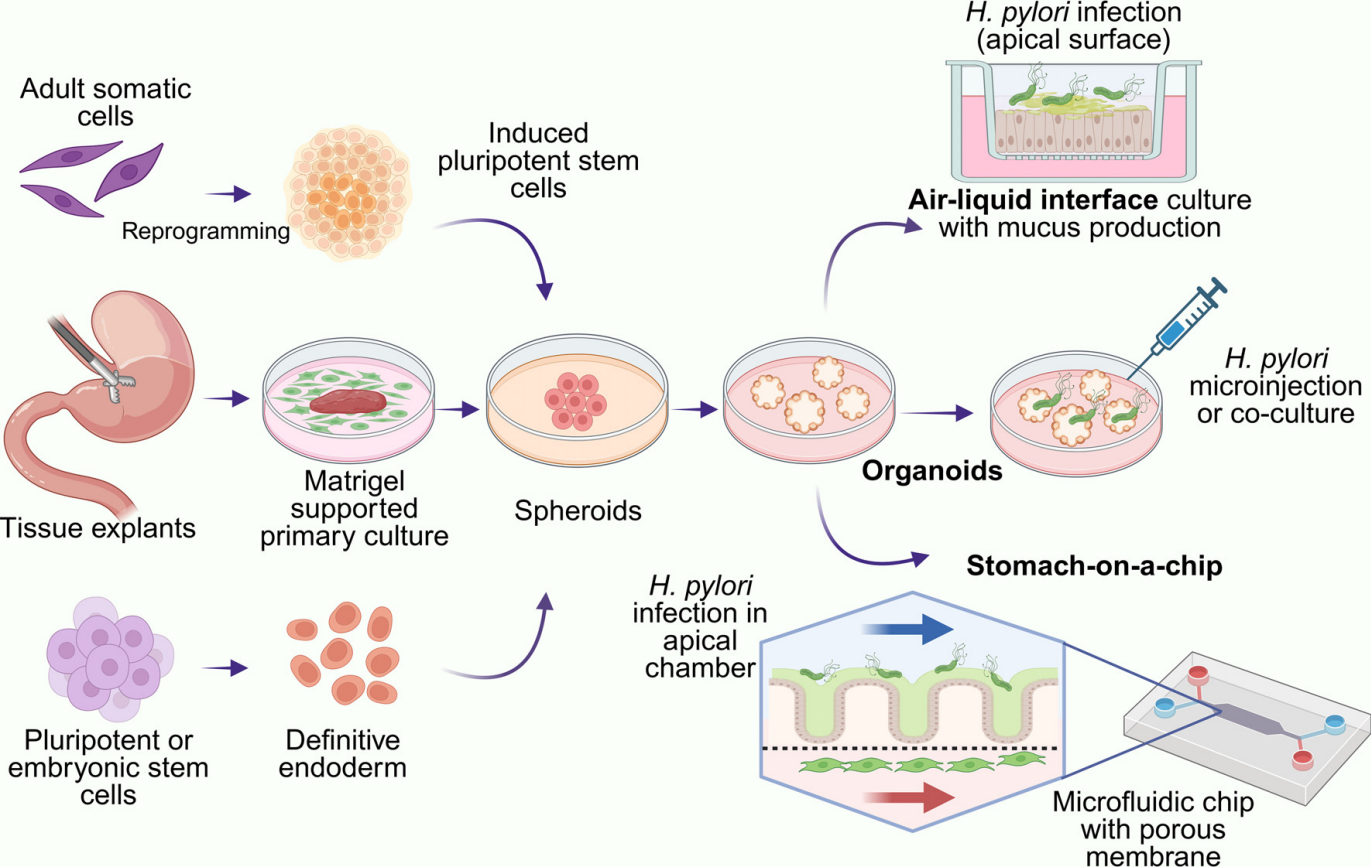
Organoid-based models of *H. pylori* infection. Gastric organoids can be derived from mouse or human tissue explants, adult somatic cells or stem cells. Created with BioRender.

Organoids are simplified, tissue-engineered *in vitro* model systems that replicate various features of the complex structures and function found in corresponding biological tissues (Chakrabarti and Zavros, 2020; Hofer and Lutolf, 2021; Idowu et al, 2022). Organoids can be generated from pluripotent or tissue-resident stem cells, whether embryonic or adult, or from differentiated cells isolated from healthy or diseased tissues (Hofer and Lutolf, 2021). More recently, organoids derived from primary cells have gained traction as suitable models for *ex vivo* studies, primarily due to their ability to closely mimic human gastrointestinal physiology. Recent advancements in gastric organoid models to study *H. pylori* infection could reduce an overdependence on ‘abnormal’ cancer cell lines and enhance the exploration of the initial stages of gastric carcinogenesis (**Figure 3**), thereby facilitating a deeper understanding of its pathogenesis (Idowu et al, 2022).

### Gastric organoids

Induced pluripotent stem cells (iPSC) are derived from adult somatic cells that undergo genetic reprogramming to attain a state akin to embryonic stem cells, achieved through the enforced expression of specific genes and factors crucial for maintaining pluripotency (Ye et al, 2013). Organoid models, a relatively recent advancement in three-dimensional (3D) cell cultivation systems, can be derived from iPSCs by controlled differentiation steps (Pellegrino and Gutierrez, 2021; Seidlitz et al, 2021). Gastric organoids can effectively mimic the cellular diversity and architectural complexity of the stomach, encompassing various epithelial cell types like mucous-secreting cells, chief cells, parietal cells, and enteroendocrine cells (Seidlitz et al, 2021). Given that the stomach serves as the primary site for *H. pylori* colonization and infection in humans, gastric organoids have emerged as invaluable tools for modeling infection and related gastric diseases such as cancer or ulcers (Ku et al., 2022). Compared to immortalized gastric cancer cell lines, organoid cultures offer a closer recapitulation of *in vivo* conditions, particularly in studying interactions between *H. pylori* and the apical-junctional complex (Uotani et al., 2019).

#### Modeling *H. pylori* infection in gastric organoids

Upon infection, gastric organoids recapitulate key aspects of *H. pylori*-induced diseases, including inflammation, epithelial damage, and dysregulation of tissue homeostasis (Idowu et al, 2022; Jeong et al., 2023; Seidlitz et al, 2021). A study by McCracken et al. demonstrated that microinjection of *H. pylori* into human iPSC-derived gastric organoids led to enhanced epithelial cell proliferation (McCracken et al., 2011). This finding further implied that *H. pylori* infection stimulates the proliferation of gastric epithelial cells, which might contribute to tissue repair mechanisms or pathological changes associated with chronic infection. During infection, CagA was injected into organoid cells via the T4SS and phosphorylated CagA bound to the Src homology 2 (SH2) domain-containing tyrosine phosphatase 2 (SHP-2) (Higashi et al, 2002). Such binding resulted in the activation of the Ras-extracellular signal-regulated kinase (Erk) signaling pathway, promoting cell proliferation, migration and survival. A similar study provided insights into the role of the Cag-SHP-2 interaction in *H. pylori*-induced gastric carcinogenesis using a similar gastric organoid model (Higashi et al, 2002).

Organoids infected with *H. pylori* exhibit alterations in glandular morphology, release of pro-inflammatory cytokines, and activation of signaling pathways associated with host defense and immune responses (Aguilar et al., 2022; Chakrabarti et al., 2021). Gastric organoid models have provided insights into the mechanisms underlying host-pathogen interactions during *H. pylori* infection, including bacterial adhesion, invasion, and modulation of host cell signaling pathways (Chakrabarti et al., 2021; Chakrabarti and Zavros, 2020). Due to the fact that organoids can be infected with *H. pylori* either by direct exposure to the bacterium or by incubation with specific bacterial components, gastric organoids have emerged as valuable tools for studying *H. pylori* infection and its pathogenesis.

### Intestinal organoids

Intestinal organoids, also known as enteroids or colonoids, are derived from intestinal stem cells and mimic the cellular composition and architecture of the intestine (Lee et al., 2018; Mahe et al., 2013; Ohki et al., 2020). Although *H. pylori* primarily infects the stomach, studies suggest that it can transiently colonize the duodenum and colon (Fujimori, 2021). A model of mouse intestinal organoids generated from isolated intestinal crypts demonstrated the role of gastric hormones in inflammation and repair thus showing that endocrine cells within these organoids closely resemble those in the gut (Ohki et al., 2020). Given the roles played by gastric hormones in *H. pylori*-associated disease manifestation, intestinal organoids can be infected with *H. pylori* to study its interaction with the intestinal epithelium and investigate potential extra-gastric effects of the infection (Becher et al., 2010; Walduck and Becher, 2012) including alterations in gut microbiota composition and gastrointestinal symptoms such as diarrhea and irritable bowel syndrome (Tan and Goh, 2012; Tohumcu et al., 2024; Cui et al., 2025).

### Co-culture of organoid and immune cells

To recapitulate the host immune response to *H. pylori* infection, researchers can co-culture organoids with immune cells such as macrophages, dendritic cells, and T cells. Co-culture systems grow stomach organoids with immune cells from the same host. This helps us learn more about the roles that innate and adaptive immune cells play in *H. pylori* infection (Idowu et al., 2022). Sebrell et al., 2019, investigated the recruitment of dendritic cells during *H. pylori* infection using human gastric organoids co-cultured with monocyte-derived dendritic cells and cataloged the various chemokines that were expressed in response to infection (Sebrell et al., 2019). The effect of *H. pylori* infection on cytokine production by innate immune cells using co-cultured gastric organoids and macrophages from infected mice has been studied as well (Suarez et al., 2019). These studies revealed a remarkable increase in cytokine production in Nod1-deficient cells, particularly when both macrophages and organoids lacked Nod1, suggesting that functional Nod1 suppresses cytokine production (Suarez et al., 2019).

Additionally, changes in mucin and antimicrobial peptide expression resulting from activation of innate and adaptive immunity have been investigated in these models. The bactericidal activity of mucus in a two-dimensional mucosoid culture model was demonstrated where it also acts as a physical barrier against *H. pylori* attachment (Boccellato et al., 2019). A co-culture of mucosoid organoids offers the potential to explore the effects of CD4+ T cell subsets and innate lymphoid cells on epithelial antimicrobial activity and offers opportunities to generate vaccinated organoids to test specific cytokines or hormone’s roles in protective responses as well as its associated mechanisms. Research employing co-culture organoids containing immune cells from the same host sheds light on the interaction between *H. pylori* and the host immune system during infection and advances our knowledge of the immunopathogenesis of diseases brought on by *H. pylori.*

#### Organoid-on-a-chip

Patient and stem cell derived gastrointestinal organoids have become an important tool for the study of *H. pylori* associated pathophysiology. Further advancements in these models have concentrated on integrating microfluidics to regulate the movement of cells, signaling molecules, and physical stimuli through channels and membranes that are bolstered by an extracellular matrix (ECM) constituent (Moses et al., 2021). These additional features introduce a significant layer of complexity, enhancing the resemblance of these models to the native structure and function of the stomach. The stomach-on-a-chip model is based on a sandwich’ structure that consists of two microchannels divided by a porous membrane to simulate the mucosal and basal surfaces of the stomach epithelium. Jeong et al., 2023 have described a human stomach micro-physiological system (hsMPS)-on-a-chip with epithelial cells derived from human antral organoids and primary mesenchymal stromal cells extracted from stomach tissue, co-cultivated under controlled flow (Jeong et al., 2023). This model recreated the maturation of gastric epithelial cells, leading to the formation of a mesh‐like mucus layer with mucus protective peptides and functional epithelial junctional complexes that exhibited gastroprotective effects against *H. pylori.* Although this model has been proposed as a platform for evaluating the antibacterial drug candidates, the porous membrane used for barrier separation can be prone to non-specific absorption of small molecules which can hinder the study. Another elegant model of the human stomach-on-a-chip demonstrated long-term growth of cannulated gastric organoids with biochemical agents delivered through the lumen using a peristaltic pump (Lee et al., 2018). This system recreated the rhythmic stretch and contraction of the organoid, reminiscent of gastric motility. Overall, with recent advances in stem cell technology and 3D matrices for supporting organotypic cultures, there is significant potential for these *ex vivo* models in identifying and validating novel therapeutic and vaccine targets (Li et al., 2022), while reducing an over-reliance on animal models.

## Advantages and limitations

### Animal models

The use of animal models has greatly advanced our understanding of the critical determinants of *H. pylori* pathology and factors involved in mediating host response to the infection, particularly the many drivers of the different phases leading to the development of gastric cancer (Ansari and Yamaoka, 2022). These models have also enabled the testing of many antimicrobials and vaccines over the years. Although early research on *H. pylori* infection was conducted mostly on non-rodent models, the isolation and generation of the mouse-adapted *H. pylori* strain has made the mouse model the cornerstone of *H. pylori* infection studies. However, there are several limitations to using mice as a surrogate for human pathophysiology and anatomy. Mouse metabolic processes are much faster compared to humans, and they can tolerate higher doses of most administered substances due to a quicker rate of kidney filtration and excretion (Sharma and McNeill, 2009). Furthermore, the mouse stomach is quite distinct from the human organ. Mice have a squamous glandular epithelium rather than the oxyntic type found in humans and typically display gastritis features within the stomach corpus as opposed to the antral gastritis observed more typically in humans, following *H. pylori* infection. Stomach cancer is also not easily modeled in this host without interfering with the expression of specific genes. Mongolian gerbils and guinea pigs have been suggested as alternatives, but they have not had the same success as the mouse models, perhaps due to the lack of molecular resources and relevant expertise.

### Non-animal models

In recent years, the complexity of non-animal models, particularly 3D organoids, has increased tremendously, putting these at par with traditional animal models. In some instances, human organoids are superlative to animal models with the added benefit of being more efficient and cost-effective. The 3Rs’ (replace, reduce and refine) objectives that underpin the ethical frameworks governing animal research in most countries today can be complemented by an increased reliance on non-animal models in the coming years. These models can further encompass *in silico* simulations, which can inform subsequent *in vitro* or animal studies, as well as advanced 2D/3D cell cultures and *ex vivo* tissue explant cultures. This review focuses specifically on the emerging popularity and application of 3D organoid models within the *H. pylori* research community.

Organoids derived from genetically modified stem cells or patient-derived iPSCs allow for the investigation of host genetic factors influencing susceptibility to *H. pylori* infection and disease outcomes. Current iPSC derived gastric organoids closely resemble the cellular composition and architecture of the stomach. Technological advances have made it possible to generate adult stem cell-derived organoids from tissues obtained from a healthy or diseased donor (Pellegrino and Gutierrez, 2021). Donor-matched organoids have diverse applications in the development of precision medicine, particularly for gastric cancer, which continues to have a poor five-year survival rate. Furthermore, gastric organoids can be adapted for high-throughput screening assays to identify novel therapeutics targeting *H. pylori* infection and associated diseases (Du et al., 2020; Lukonin et al, 2021; Li et al., 2022). Organoid-based screening platforms enable the evaluation of drug efficacy, toxicity, and mode of action in a scalable and cost-effective manner. The added dimension of immune cell co-culture allows for the investigation of both innate and adaptive immune responses to *H. pylori* infection, providing a more detailed understanding of host-pathogen interactions. This approach permits the study of immune cell recruitment, activation, and function within the context of the gastric or intestinal epithelium during *H. pylori* infection and provides opportunities to study vaccine-induced immune responses against *H. pylori*.

Obvious limitations of these models are related to the availability and accessibility to the organoid model of interest besides the difficulties pertaining to the optimization of culture conditions and the reproducibility of experimental results (Eicher et al, 2018; Pang et al., 2022). Adult stem cells and iPSCs from genetically and phenotypically well-characterized donor pools may be difficult to source. Culture media for any organoid must be carefully optimized to support the growth and differentiation of stem cells while maintaining the physiological relevance of the model (Sato et al., 2009). Achieving the appropriate balance of growth factors, signaling molecules, and nutrients is crucial for the long-term maintenance of organoid cultures, while also avoiding the common pitfall of batch variation in media components. The composition and stiffness of the extracellular matrix (ECM) used for embedding gastric organoids can influence their growth, differentiation, and functionality. Optimization of ECM components, such as Matrigel^®^ or synthetic hydrogels, is necessary to better mimic the native gastric microenvironment (Pang et al., 2022). Furthermore, most organoids lack vasculature which can lead to necrosis of underlying cells and hypoxia-induced stress responses unrelated to infection pathophysiology.

## Summary

Ever since the association between *H. pylori* infection and the development of gastritis, ulcers, and ultimately gastric cancer was first identified, considerable progress has been made in understanding the complex interplay between bacterial, host, and environmental factors that affect the course of disease. Currently, clinical presentations of *H. pylori* related gastritis and ulcers can be effectively managed with triple or quadruple antimicrobial therapy in most cases. However, the rising emergence of antibiotic resistance among *H. pylori* clinical isolates is a grave concern and gastric cancer remains a leading cause of cancer-related deaths. Despite decades of efforts and the continually elevated prevalence of the bacteria in developing parts of the worldwide, there is still no vaccine available against *H. pylori*. The eradication of *H. pylori* remains a key issue because of several factors. The emergence of antibiotic-resistant strains, complex treatment regimens combined with poor compliance, high infection and reinfection rates, and limitations in diagnostic methods all contribute to the difficulty in successfully achieving bacterial eradication. Additionally, host factors such as immune response and genetic predisposition influence the treatment outcomes. Consequently, further research is imperative to develop novel antibiotics, identify biomarkers for early detection and treatment prediction, and explore non-antibiotic therapies.

Animal models play a crucial role in advancing our understanding of *H. pylori* pathogenesis, testing new drugs, and developing vaccines. They aid in the translation of preclinical research into clinical practice by offering insights into host-pathogen interactions, bacterial virulence, and the safety and effectiveness of possible treatments. This review has summarized advanced models available to researchers for investigating new therapeutics and vaccines against *H. pylori*, addressing a significant unmet need, particularly in regions where *H. pylori* is endemic. Studies in animal models have been instrumental in dissecting the correlates of protection and make the case for targeting *H. pylori* mediated inflammation to prevent or treat the pathological outcomes rather than aiming for complete eradication of the bacteria (Sarkar et al., 2023). Despite the lack of progress on *H. pylori* vaccines due to its complex relationship with the human host, mouse and non-human primate models remain invaluable resources for further studies. On the other hand, the precise environments for studying *H. pylori* infection and pathogenesis afforded by *ex vivo* models support high-throughput screening of therapeutics while reducing the reliance on animal testing and adhering to ethical principles. Due to their advantages over conventional *in vitro* procedures in terms of polarization, longevity, amenability, and accessibility, the use of gastric organoids has increased our understanding of *H. pylori* infection. Consequently, previously unattainable in other models, key features of chemotaxis, the intracellular effects of *H. pylori* virulence factors, interactions with the apical-junctional complex, innate immune activation, and the initiation of inflammation by *H. pylori* have been unraveled using gastric organoids.

## Future perspectives

Future research on *H. pylori* infection and its associated diseases should include both animal and non-animal systems, to address current challenges and unserved needs. The development of novel therapeutics is vitally important, particularly in view of the increasing prevalence of antibiotic-resistant *H. pylori* strains. Researchers should focus on identifying new antimicrobial compounds and exploring alternative therapeutic strategies, such as bacteriophage therapy, antimicrobial peptides, and host-targeted approaches. The assimilation of precision medicine, using donor-matched organoids and advanced *ex vivo* models, can facilitate personalized treatment regimens and improve therapeutic outcomes. These models can also aid in high-throughput screening of potential therapeutics, accelerating the discovery and development process. Furthermore, considering the increasing numbers of aging populations around the world, the identification of biomarkers for early detection of gastric cancer and prediction of treatment response will be crucial in reducing the associated mortality. Therefore, continued collaboration between multidisciplinary research teams and the integration of cutting-edge technologies will be essential for overcoming the persistent challenges in *H. pylori* eradication and improving global health outcomes in the coming decades. 
